# When Steroids Backfire: A Case of Hyperosmolar Hyperglycemic State, Pneumocystis Pneumonia, and Cauda Equina Syndrome

**DOI:** 10.7759/cureus.112402

**Published:** 2026-07-10

**Authors:** Bryson T Grondel, Neel Patel, Chris Talone, Nathalie Vanderrijst

**Affiliations:** 1 Graduate Medical Education, Inspira Medical Center, Mullica Hill, USA; 2 Internal Medicine, Inspira Medical Center, Mullica Hill, USA; 3 Radiology, University at Albany, State University of New York, Albany, USA; 4 Critical Care Medicine, Cooper University Hospital, Camden, USA

**Keywords:** cauda equine syndrome, hyperosmolar hyperglycemic state (hhs), pjp pneumonia, prednisone treatment, steroid overuse

## Abstract

Corticosteroids are potent anti-inflammatory and immunosuppressive agents frequently used in short courses for various conditions. However, prolonged or inappropriate use can lead to severe complications, including immunosuppression, metabolic derangements, and delayed diagnosis of underlying disease. Cauda equina syndrome is a neurosurgical emergency that can be masked by corticosteroid therapy, while chronic use also predisposes to opportunistic infections such as *Pneumocystis jirovecii* pneumonia. We present a case of corticosteroid-induced hyperosmolar hyperglycemic state, *Pneumocystis jirovecii* pneumonia, and cauda equina syndrome, illustrating the systemic consequences of unsupervised steroid use.

## Introduction

The potent anti-inflammatory and immunosuppressive effects of corticosteroids make them useful for short courses of therapy across a wide range of inflammatory, allergic, autoimmune, and neurologic conditions. However, prolonged use is associated with numerous adverse effects. In a landmark meta-analysis of 93 studies, including 6,602 patients, Conn and Poynard found that chronic corticosteroid use was associated with a higher prevalence of diabetes, osteoporosis, and sepsis [1].

Corticosteroids disrupt multiple mechanisms involved in glucose homeostasis [1,2]. Angelopoulos et al. described this process as multifactorial, involving acute hyperglycemia, insulin resistance, and, in some patients, the development of new-onset steroid-induced diabetes [2]. Acutely, steroid-induced hyperglycemia is driven by a reduction of insulin-mediated glucose uptake into skeletal muscle, increased hepatic glucose production through glycogenolysis, and gluconeogenesis driven by protein catabolism. Over time, corticosteroids lead to peripheral insulin resistance and impaired pancreatic beta cell function, thus resulting in new-onset diabetes. Steroid-induced diabetes is estimated to occur in 10%-20% of patients receiving chronic glucocorticoid therapy and may develop insidiously in patients without a prior history of hyperglycemia [2]. In severe cases, this metabolic derangement may progress to hyperosmolar hyperglycemic state, a life-threatening endocrinologic emergency characterized by profound hyperglycemia, hyperosmolality, and dehydration [3,4].

In patients with underlying inflammatory, degenerative, or neuromuscular disorders, corticosteroids may also suppress pain and inflammatory symptoms, potentially delaying recognition of progressive disease. For example, patients with degenerative disc disease and lumbar spinal stenosis may develop radicular symptoms that, if unrecognized or untreated, can progress to cauda equina syndrome. Cauda equina syndrome is a rare but serious neurologic emergency caused by compression of the lumbosacral nerve roots that form the cauda equina [5]. It commonly presents with severe back pain and red flag symptoms, including saddle anesthesia, bowel or bladder dysfunction, sensory deficits, and radicular pain [5,6]. Prompt recognition and surgical intervention are critical to prevent permanent neurologic damage [5-7].

Chronic corticosteroid use is also associated with acquired immunosuppression due to multiple mechanisms. These include, but are not limited to, downregulation of transcription of inflammatory cytokines, inhibition of leukocyte function (migration, translocation, and activation), and lymphocyte apoptosis [8,9]. This immunosuppression increases the risk of infections, including opportunistic infections, and worse clinical outcomes [1,8-14]. Patients with reduced innate and cell-mediated immune function are at increased risk of acquiring this infection. *Pneumocystis jirovecii* pneumonia is a potentially life-threatening opportunistic infection that often presents with non-specific symptoms, including dyspnea, fever, and cough [11-14]. These symptoms may overlap with other respiratory, metabolic, or inflammatory conditions, making timely diagnosis and treatment challenging.

This case report describes a rare constellation of clinical findings associated with the adverse effects of prolonged steroid use. Herein, we present the case of a patient with chronic, unsupervised corticosteroid use who presented with overlapping features of hyperosmolar hyperglycemic state, cauda equina syndrome, and *Pneumocystis jirovecii* pneumonia. This case highlights the diagnostic and therapeutic challenges of managing concurrent endocrine, neurologic, and infectious emergencies in the setting of chronic immunosuppression and masked symptoms.

## Case presentation

A 67-year-old male with chronic obstructive pulmonary disease, cervical myelopathy, chronic low back pain, and hypertension presented with polyuria, polydipsia, dyspnea, and altered mental status. On arrival, the patient was afebrile, hemodynamically stable, non-tachypneic, and saturating well (SpO_2_: 96%-100%) on room air. Physical examination revealed an agitated, confused male who was alert and oriented to person, place, and time but demonstrating aggression towards staff. His wife stated that he typically had a calm demeanor, and this significant change in his behavior had prompted their presentation to the emergency department. Examination was also notable for dry mucous membranes and skin tenting but was otherwise unremarkable. Laboratory studies revealed a serum glucose of 899 mg/dL, a serum osmolality of 340 mOsm/kg, a bicarbonate of 24.3 mmol/L, and the absence of ketonuria or ketonemia. Subsequent HbA1c revealed a value of 10.8%. Together, these findings were consistent with newly diagnosed diabetes mellitus presenting as acute hyperosmolar hyperglycemic state, supported by marked hyperglycemia, clinical evidence of dehydration, and acute metabolic encephalopathy.

He was admitted to the intensive care unit for intravenous insulin infusion and fluid resuscitation. Upon further discussion with the patient, he disclosed that he had been taking 40 mg of prednisone daily for the past eight months. The initial prescription had been given for an acute exacerbation of chronic neck and low back pain with radicular symptoms. This led to complete resolution of his symptoms, so he requested refills from multiple providers at different clinics and different health systems. He was given multiple prescriptions for prednisone 5 mg with refills. Available pharmacy records demonstrated that he had received more than 1,800 tablets during this eight-month time period. Based on patient report and chart review, the patient was unaware of the risks of prolonged use, and there was no ongoing monitoring of his chronic corticosteroid use because he was requesting refills from different providers in different health systems. Despite prescriptions being written with specific instructions for short durations and tapers, he collected the tablets and reported self-administering eight tablets (40 mg) daily.

**Figure 1:**
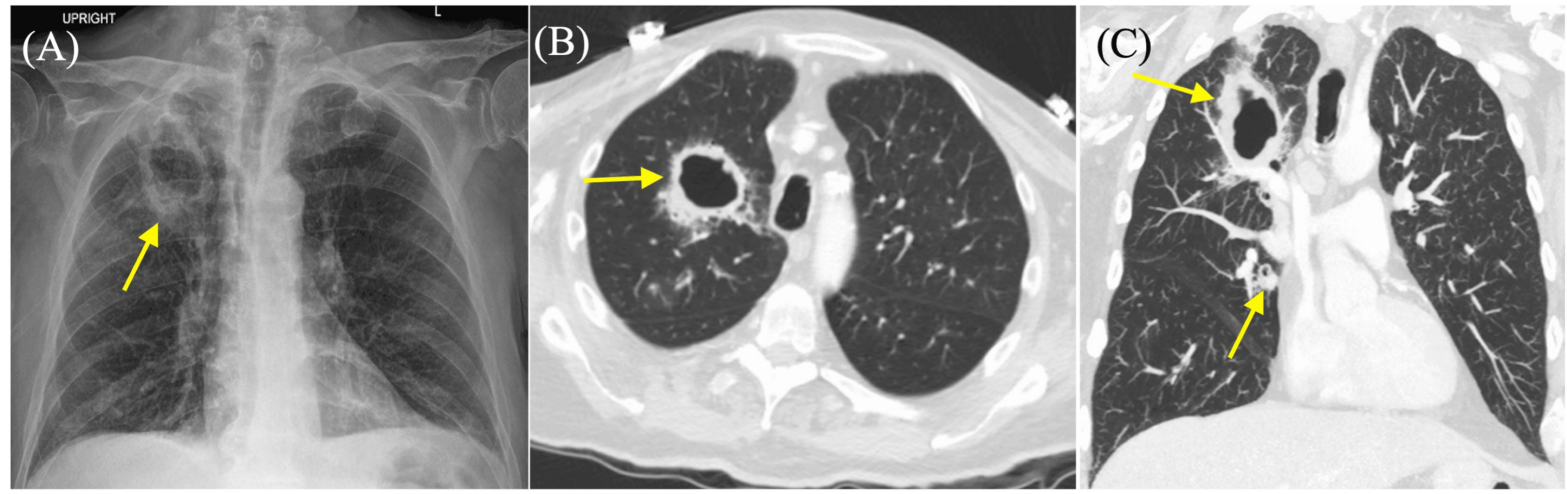
Radiography and computed tomography of the thorax (A) AP chest X-ray showing a cavitary lesion in the right upper lobe measuring ~6 cm. (B) CT chest (coronal) showing multiple cavitating nodular consolidations within the right upper lobe, measuring up to 4.4 x 3.8 cm in the right suprahilar region, with adjacent ill-defined ground-glass opacity. (C) CT chest (axial) showing a large cavitary lesion in the right upper lobe with surrounding inflammatory changes. AP, anteroposterior; CT, computed tomography.

After initial stabilization, chest imaging revealed new cavitary lesions with associated ground-glass opacities and clustered tree-in-bud opacities within the right upper lobe (Figures 1A-1C). Presentation and findings were concerning for an atypical infectious process. The patient’s dyspnea and altered mental status resolved after resolution of the hyperosmolar hyperglycemic state, and he did not report or demonstrate symptoms of acute respiratory infection, so antibiotics were held pending further investigation. However, on hospital day five, he developed a fever and new tachycardia, so he was started on intravenous antibiotics -- ceftriaxone (1 g daily) and azithromycin (500 mg daily). The patient was unable to produce sufficient sputum to send for culture. To further evaluate the cavitary lesion and obtain cultures, he was taken for bronchoscopy and bronchoalveolar lavage. Cultures from the bronchoscopy showed light growth of pan-sensitive *Pseudomonas aeruginosa*, moderate growth of *Candida albicans*, light growth of Paecilomyces, negative acid-fast stain, and positive PCR for *Pneumocystis jirovecii*. The patient's history was negative for tuberculosis risk factors, and QuantiFERON-TB Gold was negative. Testing for immunocompromising conditions, including HIV, hepatitis B, and hepatitis C, was also negative.

Cultures obtained during bronchoscopy were finalized before PCR results were available, so the patient was started on cefepime (2 g every eight hours). After *Pneumocystis jirovecii* was detected on PCR, oral sulfamethoxazole-trimethoprim (800-160 mg, two tablets every eight hours) was added. Given the indolent presentation and lack of respiratory symptoms, it was suspected that Pneumocystis was the primary infectious agent. However, co-infection could not be ruled out, so both antibiotics were continued.

**Figure 2:**
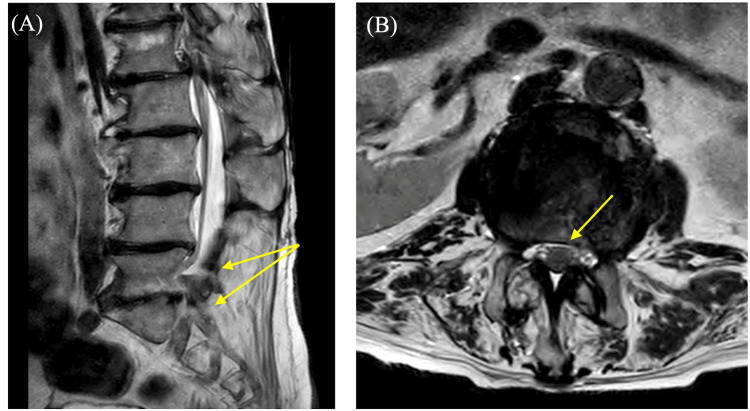
Magnetic resonance imaging of the lumbar spine (A) Sagittal and (B) axial views show severe lumbar spinal stenosis, hypertrophy of the ligamentum flavum, and compression of the thecal sac and cauda equina structures at the L5-S1 level.

Due to concern for steroid-induced immunosuppression and steroid-induced diabetes, a steroid taper was initiated on admission (30 mg daily for three days, followed by 20 mg for three days, and then 10 mg for three days before discontinuing). Shortly after taper initiation, he developed new upper and lower extremity weakness (4/5 strength bilaterally) with paresthesias and bilateral groin pain. Magnetic resonance imaging (MRI) of the lumbar spine (Figures 2A, 2B) revealed severe multilevel degenerative changes and findings consistent with cauda equina syndrome. MRI of the cervical spine also showed worsening of his known cervical myelopathy. The patient was emergently taken to the operating room and underwent C4-T1 decompression and fusion and T12-L2 laminectomy. He tolerated the procedures well but developed a wound infection postoperatively, which required surgical revision. Because of postoperative neurologic and infectious complications, he was transferred to a tertiary center for advanced neurosurgical intervention.

## Discussion

Corticosteroids are potent anti-inflammatory and immunosuppressive medications with a wide range of clinical applications. When chronic therapy is indicated, patients should be counseled on the risks of prolonged corticosteroid use, including serious and potentially life-threatening adverse effects [1]. In this case, prolonged disruption of glucose homeostasis and insulin resistance likely contributed to steroid-induced diabetes, while chronic immunosuppression predisposed the patient to opportunistic infection [1,2,4,8-14]. Adding to the novelty of this case is the finding of a cavitary lesion in a patient with *Pneumocystis jirovecii* pneumonia. Though rare, a number of case reports have documented the presence of cavitary lesions in patients with *Pneumocystis jirovecii* pneumonia [12,14-17]. It should be noted that we were unable to rule out co-infection with Pseudomonas, but the patient's indolent presentation, immunocompromised status, and overall clinical picture were more consistent with Pneumocystis pneumonia.

His presentation with hyperosmolar hyperglycemic state was likely multifactorial in the setting of undiagnosed diabetes mellitus and chronic steroid use and was likely precipitated by the underlying respiratory infection. Additionally, his postoperative course was complicated by a surgical site infection, likely related in part to impaired wound healing. Wang et al. reported that chronic corticosteroid use can impair wound healing by disrupting the inflammatory, proliferative, and remodeling phases through mechanisms that compromise fibroblast function, reduce collagen tensile strength, and alter the local cytokine milieu [18,19].

Although steroid-induced diabetes, acquired immunosuppression, and wound complications are well established in the literature, this case also illustrates a less commonly emphasized consequence of chronic corticosteroid use: the potential masking of neurologic or musculoskeletal symptoms and progression, or even acceleration, of underlying pathologic conditions [1,2,5-8,20]. The patient had a known history of cervical myelopathy (per patient, confirmed on MRI of the cervical spine -- we were unable to retrieve these images) and low back pain (advanced imaging had not previously been completed), but he had not pursued physiatric or neurosurgical evaluation because he reported complete symptomatic relief with daily prednisone use. He also reported intermittent low back pain before initiating corticosteroids but denied prior radicular symptoms in his lower extremities. In retrospect, the progression of cervical myelopathy and lumbar degenerative disc disease may have been underrecognized because of the chronic anti-inflammatory and analgesic effects of prednisone therapy. Because the patient had not completed osteoporosis screening before admission, it was not possible to determine whether reduced bone mineral density contributed to disease progression or postoperative complications. However, it is also possible that chronic steroid use may have accelerated the progression of his underlying disease through demineralization and compromise of the involved vertebral bodies. This association may warrant further investigation, particularly in patients receiving prolonged corticosteroid therapy for chronic pain or inflammatory symptoms.

## Conclusions

Chronic corticosteroid use, particularly when unsupervised or prolonged, can contribute to life-threatening endocrine, infectious, and postoperative complications while also masking symptoms of underlying neurologic or musculoskeletal disease. Clinicians should exercise vigilance when prescribing and monitoring corticosteroid therapy, including appropriate tapering, refill oversight, and patient education regarding the risks of unsupervised use. This case underscores the importance of a multidisciplinary approach to recognizing, preventing, and managing the systemic sequelae of chronic corticosteroid exposure.
